# Mesenchymal stem cell-derived extracellular vesicles: emerging cell-free therapeutics for kidney diseases

**DOI:** 10.3389/fimmu.2026.1738491

**Published:** 2026-03-04

**Authors:** Si-Jie Chen, Tao-Tao Tang, Yi-Lin Zhang, Lin-Li Lv, Bi-Cheng Liu

**Affiliations:** Institute of Nephrology, Zhongda Hospital, School of Medicine, Southeast University, Nanjing, Jiangsu, China

**Keywords:** cell-free, extracellular vesicles, kidney diseases, mesenchymal stem cells, therapeutics

## Abstract

The increasing global impact of kidney diseases highlights a pressing and unmet need for new treatment strategies. In this context, mesenchymal stem cell-derived extracellular vesicles (MSC-EVs) have emerged as a promising cell-free therapeutic approach, attracting growing interest due to their dual regenerative functions. MSC-EVs not only possess intrinsic therapeutic effects mediated by their cargo of mRNAs, proteins, and miRNAs that regulate inflammation, promote cell repair, reduce fibrosis, but also serve as highly biocompatible vehicles for drug delivery. This versatility places them at the forefront of a potential shift in how kidney diseases may be treated. In this review, we systematically summarize current knowledge on the mechanisms and therapeutic potential of MSC-EVs in various kidney diseases. Although challenges related to standardization and clinical translation persist, ongoing progress supports the view that MSC-EVs are poised to become key next-generation therapies for kidney diseases.

## 1 Introduction

The increasing global prevalence of kidney diseases, including both acute kidney injury (AKI) and chronic kidney disease (CKD), presents a serious and growing public health challenge. AKI is characterized by a rapid and often severe decline in kidney function. However, current treatment options remain highly limited, and delayed diagnosis is associated with mortality rates reaching as high as 50% (1). For patients who survive the initial episode, long-term outcomes remain concerning, with a substantially increased risk of developing CKD, a progressive and irreversible condition that already affects nearly 10% of the global population (2). In China alone, the estimated number of CKD patients is about 130 million, highlighting the magnitude of this under recognized epidemic (3, 4). Available therapies for these kidney diseases are largely insufficient and do not effectively prevent disease progression. Furthermore, long-term use of immune-suppressants or dependence on renal replacement therapy often leads to significant systemic side effects. Therefore, there is a pressing and unmet need for new treatment strategies that can promote true kidney repair while minimizing off-target toxicity.

In response to this clinical challenge, regenerative medicine has emerged as a promising approach. Mesenchymal stem cells (MSCs) were initially regarded as a potential strategy for tissue regeneration due to their ability to differentiate into various cell types (5). However, a major shift in perspective has reshaped this view: rather than relying on MSCs engraftment, it is now understood that their potent paracrine signaling mediated by a diverse set of secreted factors plays the central role in promoting kidney repair (6, 7). This insight has elevated extracellular vesicles (EVs) as a key focus in nephrology research. EVs are natural, lipid-bound nanoparticles that carry a variety of bioactive molecules, including proteins, nucleic acids, and lipids, and serve as critical mediators of cell-to-cell communication (8, 9). Based on differences in biogenesis pathways, size, and markers, they are primarily categorized into three major subtypes: Exosomes, typically ranging from 30 to 150 nanometers in diameter, originate from the fusion of intracellular multivesicular bodies with the plasma membrane upon release. Microvesicles, approximately 100 to 1000 nanometers in diameter, are formed by direct outward “budding” from the plasma membrane. Apoptotic bodies, usually larger than 1 micrometer in diameter, are produced during the process of apoptosis. A growing body of evidence suggests that EVs, especially those released by MSCs, significantly contribute to reducing injury and slowing disease progression in both AKI and CKD, thus revealing a promising new therapeutic pathway (10, 11).

The unique advantage of MSC-EVs stems from their capacity to replicate the diverse therapeutic effects of the parent cells. They effectively coordinate anti-inflammatory, pro-repair, anti-fibrotic, and immunomodulatory activities, while avoiding the risks of tumor formation and immune rejection linked to whole-cell transplantation (12, 13). This makes MSC-EVs a favorable cell-free therapeutic approach. In addition to their intrinsic biological properties, their natural biocompatibility and targeting ability have motivated efforts to engineer them into advanced drug delivery systems. By loading these vesicles with exogenous therapeutic agents, their innate regenerative functions can be enhanced, offering broad potential for targeted molecular treatments in kidney disease and other conditions (14, 15).

This review aims to synthesize the rapidly advancing field of MSC-EVs as therapeutics for kidney diseases. We will critically examine the mechanistic basis for their efficacy, assess their application across diverse kidney diseases, and discuss the translational challenges that must be overcome to harness the full potential of these nanoscale mediators in reshaping the future of nephrology.

## 2 Mechanisms of MSC-EVs in treating kidney diseases

**Figure 1:**
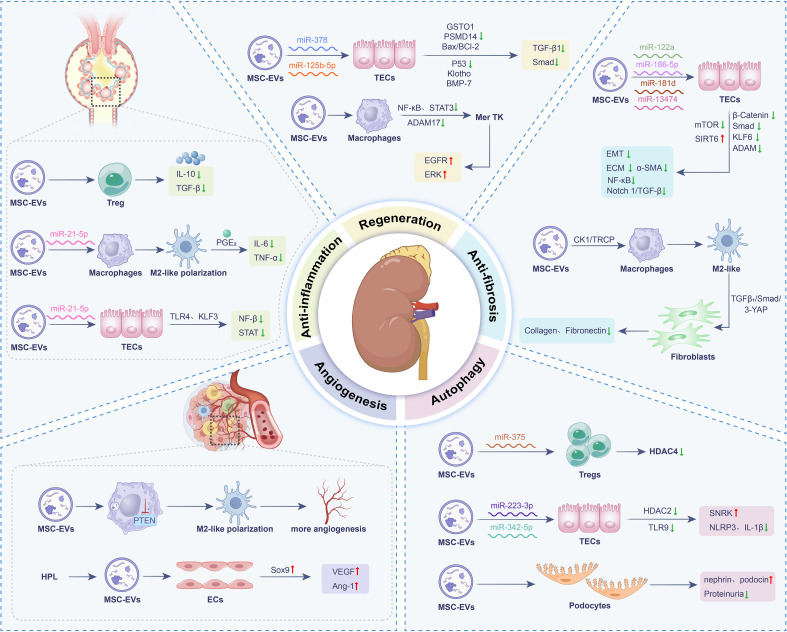
Mechanism schematic of MSC-EVs in the treatment of kidney diseases. Bone morphogenetic protein (BMP), endothelial cells (ECs), epithelial-mesenchymal transition (EMT), extracellular matrix (ECM), glutathione transferase Omega 1 (GSTO1), interleukin (IL), Krüppel-like factor 3 (KLF3), matrix metalloproteinases (MMPs), mesenchymal stem cell-derived extracellular vesicles (MSC-EVs), microRNAs (miRNAs), NACHT, LRR and PYD domains-containing protein 3 (NLRP3), nuclear factor κ-light-chain-enhancer of activated B cells (NF-κB), prostaglandin E2 (PGE2), proteasome 26S subunit, non-ATPase (PSMD), regulatory T cells (Tregs), signal transducer and activator of transcription (STAT), silent information regulator transcript (SIRT), tubular epithelial cells (TECs), tumor necrosis factor-α (TNF-α), vascular endothelial growth factor (VEGF).

Despite heterogeneity in cargo composition due to differences in cellular origin and microenvironment, MSC-EVs exert their therapeutic effects through conserved mechanisms of intercellular communication (16). The primary action modes encompass three pivotal pathways: receptor-ligand interactions on target cell surfaces to modulate intracellular signaling; direct fusion with the plasma membrane to deliver membrane associated proteins and lipids; and endocytosis or targeted delivery to transfer bioactive molecules including proteins, lipids, and nucleic acids into recipient cells, thereby reprogramming cellular functions (17, 18) (**Figure 1**).

### 2.1 Anti-inflammatory and immunomodulatory effects

In AKI or CKD, excessive or persistent inflammation drives death, fibrosis, and functional decline of renal cells. Controlling inflammation is therefore essential not only for alleviating acute damage but also for preventing progression to end stage renal disease. MSC-EVs mediate a multifaceted suppression of renal inflammation through synergistic mechanisms (19). They carry specific miRNAs, which suppress key pro-inflammatory signaling pathways. At the same time, immunomodulatory cargo molecules like interleukin (IL)-10 and transforming growth factor β (TGF-β) enhance the expansion and functional maturation of regulatory T cells (Tregs). In addition, surface enzymes including CD73 generate adenosine via catalytic activity, and subsequent adenosine receptor signaling helps to suppress excessive immune activation. Collectively, these coordinated actions potently reduce renal inflammation and alleviate tissue injury (20).

In murine models of renal ischemia-reperfusion injury (IRI), MSC-EVs consistently modulate the cytokine balance toward an anti-inflammatory profile, marked by reduced levels of IL-6 and tumor necrosis factor (TNF) -α and increased IL-10, thereby improving the inflammatory microenvironment (21). EVs derived from bone marrow MSCs (BMSCs) overexpressing indoleamine 2,3-dioxygenase (IDO) promote M2 macrophage polarization, enhance the proliferation of tubular epithelial cells (TECs), and suppress apoptosis and fibrosis, all of which contribute to accelerated renal repair (22). Similarly, Yao et al. reported that EVs released by IL-1β pre-conditioned MSCs are enriched with miR-21, which strongly induces M2 polarization and improves survival in septic mice (23). In addition to immunomodulation, MSC-EVs help maintain mitochondrial homeostasis by restoring the expression of transcription factor A (TFAM), thereby preventing mitochondrial DNA leakage and subsequent inflammatory activation in injured kidneys (24).

Adipose derived MSC-EVs (AMSC-EVs) also exhibit considerable therapeutic promise. They promote hyperpolarization of M2 macrophages and trigger a transcriptomic signature associated with prostaglandin E2 (PGE2), leading to amelioration of glomerulonephritis (25). In a study by Bian et al., AMSC-EVs were shown to transfer miR-21-5p to TECs, thereby suppressing the TLR4/NF-κB/NLRP3 pathway and attenuating inflammation and apoptosis in AKI (26). In the context of diabetic kidney disease (DKD), MSC-EVs serve as cell-free carriers that deliver RBMX packaged miR-23a-3p to human kidney proximal tubular (HK-2) cells under high glucose (HG) conditions. This mechanism involves targeting Krüppel-like factor 3 (KLF3) and inhibiting STAT3 signaling, ultimately mitigating renal fibrosis (RF) and inflammation (27). Beyond miRNAs, EVs can deliver proteins. Studies have shown that the stimulator of interferon genes (STING) can be packaged into EVs and secreted upon immune activation, thereby modulating responses in recipient cells (28). In kidney diseases, DNA released from injured renal tubules activates the cGAS/STING pathway in macrophages, driving inflammation and fibrosis (29). EVs from hematopoietic MSCs attenuate inflammation in a unilateral ureteral obstruction (UUO) model, potentially through upregulation of silent information regulator transcript 6 (SIRT6) and suppression of β-catenin signaling (30). These findings highlight the functional plasticity of MSC-EVs and their potential for therapeutic enhancement.

### 2.2 Promoting renal cell regeneration and repair

Current clinical strategies for treating AKI and CKD primarily focus on controlling risk factors to slow disease progression, yet effective methods to promote functional recovery following nephron damage remain lacking. Therefore, directly activating the intrinsic regenerative repair mechanisms has emerged as a critical direction for achieving disease-modifying therapies (31). In this regard, MSC-EVs exhibit significant potential to promote regeneration in TECs, vascular endothelial cells, and others. Key mechanisms include the delivery of miRNAs, which activate signaling pathways to drive cell cycle progression; the transfer of regeneration-associated mRNAs, such as vascular endothelial growth factor (VEGF), directly enhancing cell proliferation and repair (32).

Our group previously identified through sequencing that human umbilical cord (UC)-MSCs derived EVs are rich in miR-125b-5p, which can inhibit TECs apoptosis and cell cycle arrest by targeting p53, thereby promoting renal repair after AKI (33). Also, UC-MSC-EVs alleviate IRI *in vitro* and *in vivo* through a dual-phase mechanism: acutely inhibiting apoptosis via the Bax/Bcl-2 pathway, and chronically delaying TECs senescence via the Ras/pERK/Ets1/p53 axis. Combined with senolytics, MSC-EVs synergistically target both apoptosis and senescence, suggesting a promising strategy for improving kidney transplantation outcomes (34). In addition, Lei et al. demonstrated UC-MSC-EVs reverse BMSCs senescence by transferring proliferating cell nuclear antigen (PCNA), reducing senescence markers, enhancing self-renewal, and lengthening telomeres. They also improve bone regeneration, wound healing, and angiogenesis (35). Another study found that EVs from three-dimensional (3D) cultured MSCs exhibited significantly enhanced expression of miR-93-5p and were superior to 2D cultured EVs in treating IRI, more effectively improving function, reducing injury, and decreasing apoptosis and inflammation (36). 3D culture produces EVs with enhanced efficacy and a richer cargo profile. Simultaneously, by mimicking a more *in vivo*-like environment, it establishes a foundational process for the scalable and standardized production of high-quality therapeutic products, thereby significantly accelerating clinical translation.

Moreover, in the early stages of IRI, AMSC-EVs correct mitochondrial redox imbalance in TECs by activating the HO-1/Nrf2 antioxidant pathway. This stabilizes mitochondrial function and membrane potential, thereby reducing apoptosis of TECs. This antioxidative effect establishes a crucial metabolic foundation for cell survival and subsequent repair (37). Studies also indicate that MSC-EVs can deliver miR-378 to target PSMD14, inhibiting the TGF-β1/Smad2/3 signaling pathway, thereby mitigating fibrosis and promoting structural recovery of the renal tissue (38). Also, BMSC-EVs enriched with miR-186 target the transcription factor SRY-box transcription factor (SOX) 4 to inhibit fibroblast activation in pulmonary fibrosis (39). SOX4 is also pivotal in renal fibrosis, where it enhances the TGF-β1/Smad3 pathway and promotes extracellular matrix deposition (40). This demonstrates that EVs, as natural carriers, not only transmit signaling molecules but also intervene in pathological processes like fibrosis by modulating transcriptional networks.

### 2.3 Anti-fibrotic effects

RF represents a common and final pathological outcome in the progression of CKD, which essentially results from abnormal repair responses following long-term kidney injury (41). Studies have shown that MSC-EVs can delay this process through multiple mechanisms, thereby exerting renal protective effects. For instance, they downregulate the expression of pro-fibrotic genes, inhibit epithelial mesenchymal transition (EMT), alleviate tubular atrophy and local inflammatory responses, and furthermore promote angiogenesis and tissue repair, ultimately reducing glomerulosclerosis and RF (8). Specifically, the anti-fibrotic mechanisms of MSC-EVs mainly involve the following three aspects: inhibiting signaling pathways such as TGF-β/Smad, thereby decreasing deposition of extracellular matrix (ECM); delivering matrix metalloproteinases (MMPs), facilitating the degradation of abnormally accumulated matrix; modulating fibroblast activation by suppressing their transformation into myofibroblasts (42). In summary, through multi-pathway synergistic actions, MSC-EVs demonstrate significant potential therapeutic value in intervening in RF (43).

Studies have found that pluripotent stem cell-derived MSCs (PSC-MSCs)-EVs not only concentration dependently inhibit EMT in NRK-52E cells but also alleviate RF, suppress inflammation, and improve renal function in UUO mouse model. Further mechanistic investigation suggested that these protective effects may be associated with upregulation of SIRT6 expression and simultaneous inhibition of Wnt/β-catenin pathway. Using single-cell RNA sequencing and other technologies, one study revealed TGF-β1^+^ Arg1^+^ macrophages promote DKD fibrosis by activating the TGF-β1/Smad2/3–YAP axis in mesangial cells, inducing myofibroblast differentiation. MSC-EVs counteract this by promoting YAP ubiquitination and degradation via CK1δ/β-TRCP, disrupting the fibrotic pathway (44). In both TGF-β1-stimulated NRK-52E cells and a UUO mouse model, miR-186-5p is downregulated during RF. MSC-EVs deliver miR-186-5p to TECs, where it directly target and suppress Smad5, thereby reducing ECM deposition, EMT, and apoptosis, and alleviating RF (45). Collectively, these findings illuminate the multi-faceted mechanisms by which MSC-EVs combat RF, offering promising avenues for future therapies.

Equally compelling is the evidence for MSC-EVs as carriers of specific miRNAs that directly target and dismantle core pro-fibrotic circuits. MSC-EVs derived miRNA-122a reduce fibrosis in TECs via inhibiting mTOR, thereby ameliorating renal injury. This highlights miRNA-122a as a key anti-autophagic mediator and a potential RF therapy (46). Also, BMSC-EVs deliver miR-181d to target KLF6, inhibiting NF-κB signaling and reducing fibrosis markers (e.g. collagen I, Col4α1), thereby attenuating fibrosis progression (47). Additionally, Shi et al. found that miR-13474 enriched human UC-MSC-EVs inhibit ADAM17, suppressing the Notch 1/TGF-β pathway to reduce collagen deposition and RF. Functional studies confirmed that miR-13474 overexpression enhances this anti-fibrotic effect, whereas its knockdown diminishes it, highlighting a novel therapeutic strategy (48).

### 2.4 Angiogenesis and microcirculation improvement

Progressive decline in the density of functional microvessels, particularly peritubular capillaries is a characteristic in kidney diseases progression. This process is primarily driven by persistent microenvironmental injuries such as ischemia, hypoxia, hyperglycemia, and proteinuria, which lead to endothelial cell dysfunction, senescence, and even apoptosis. Concurrently, there is a reduction in pro-angiogenic signals like VEGF and an increase in anti-angiogenic signals, resulting in an insufficient capacity for new vessel formation to compensate for vascular loss (49). The ensuing vascular rarefaction further exacerbates local hypoxia and oxidative stress, creating a vicious cycle. Moreover, it activates hypoxia-induced pro-fibrotic pathways, ultimately promoting RF. On the other hand, MSC-EVs can repair vessels by enriching pro-angiogenic factors, carring pro-angiogenic miRNAs, modulating the function of endothelial progenitor cells, thereby significantly improving microcirculation (50).

Our previous research demonstrated that human platelet lysate (HPL) enhances production and function of MSC-EVs. Multi-omics confirmed that HPL-EVs possess a unique, potent pro-angiogenic profile, which effectively mitigates renal microvascular rarefaction and promotes endothelial regeneration (51). In a pig model of renal vascular disease (RVD), MSC-EVs surpass standard percutaneous transluminal renal angioplasty (PTRA) in improving renal function by more effectively restoring microvasculature and mitigating inflammation, oxidative stress, and fibrosis (52). Yu et al. found that MSC-EVs promote post−injury renal angiogenesis and repair through a dual synergistic mechanism. Directly, they are taken up by vascular endothelial cells, upregulating pro−angiogenic genes such as VEGFA and HIF−1α to drive endothelial proliferation, migration and tube formation. Indirectly, they activate Sox9^+^ renal progenitor cells to suppress oxidative stress and reduce α−SMA−mediated fibrosis, thereby reshaping the tissue microenvironment to favor angiogenesis (53). Another study showed that both human UC−MSC−EVs and cord−blood CD133^+^−EVs improve renal function in CKD, evidenced by elevated serum albumin (indicating reduced inflammation and improved nutrition) and decreased α-SMA (reflecting inhibited myofibroblast activation and fibrosis), collectively establishing a pro-angiogenic microenvironment (54).

### 2.5 Regulation of autophagy and pyroptosis

Autophagy, as a cellular self-protection mechanism, maintains intracellular homeostasis by degrading damaged organelles and abnormal proteins, thereby playing a protective role in both AKI and CKD. In contrast to other forms of cell death, pyroptosis is initiated by inflammatory caspases. This process triggers a massive inflammatory response that contributes to subsequent tissue injury and renal function deterioration. Autophagy can inhibit pyroptosis, and the imbalance between these two processes drives the progression of kidney disease. MSC-EVs exert dual therapeutic benefits in kidney diseases by modulating cellular autophagy and pyroptosis. On one hand, they deliver functional molecules to upregulate autophagy, helping renal cells clear damaged proteins and organelles, maintain cellular homeostasis, and inhibit apoptosis, thereby alleviating tubular injury and delaying fibrosis. On the other hand, MSC-EVs suppress hyperactivated pyroptosis pathways, reducing the release of proinflammatory cytokines and mitigating inflammatory renal injury and immune dysregulation. This synergistic action of enhancing protective autophagy while inhibiting destructive pyroptosis effectively improves renal tissue repair, suppresses fibrotic progression, and ultimately promotes renal functional recovery, offering a novel mechanism and strategy for cell-free therapy in kidney diseases. Therefore, regulating the balance between autophagy and pyroptosis is of great importance for kidney diseases (55).

A study revealed that BMSC-EVs deliver miR-223-3p, which downregulates HDAC2 to enhance SNRK transcription, thereby suppressing TECs apoptosis, inflammation, and pyroptosis to ameliorate kidney injury (56). Similarly, in a septic AKI mouse model, UC-MSCs-EVs were found to deliver miR-375 to CD4+ T cells, where this miRNA negatively regulates HDAC4 expression, thereby inhibiting lipopolysaccharide-induced apoptosis and promoting autophagy in CD4+ T cells. This can protect renal structure and function, delay or ameliorate sepsis-induced kidney injury by inhibiting apoptosis, mitigating inflammatory responses, and maintaining immune homeostasis (57). UC-MSC-EVs markedly alleviate renal histopathology in IRI, including tubular dilation, vacuolization, and collagen deposition. *In vitro*, they protect NRK-52E cells from cisplatin-induced injury. Mechanistically, UC-MSC-EVs downregulate proteins associated with fibrosis (fibronectin, α-SMA, vimentin) and NLRP3 inflammasome activation (NLRP3, caspase-1, IL-1β, GSDMD), which are elevated in IRI kidneys and cisplatin-injured cells. Another study found that serum level of miR-342-5p is downregulated while TLR9 expression is upregulated in AKI patients. It confirmed that miR-342-5p-EVs secreted by AMSCs could target and inhibit TLR9 expression, subsequently activating autophagy and mitigating LPS-induced inflammatory responses and renal injury in TECs (58). These findings confirm MSC-EVs as a multi-effective platform alleviating apoptosis, pyroptosis, and inflammation in injured kidneys.

Besides apoptosis and pyroptosis, necroptosis, a regulated form of necrotic cell death, plays a key role in pathological conditions such as acute kidney injury. EVs can modulate necroptosis by delivering bioactive molecules to recipient cells. For example, in micronodular lung cancer, serum EVs enriched in miR-412-3p suppress TEAD1 expression, promoting malignancy (59). TEAD1 is crucial for mitochondrial homeostasis and necroptosis inhibition. In cisplatin-induced kidney injury, TEAD1 upregulation protects TECs, whereas its loss exacerbates mitochondrial dysfunction and necroptosis via RIP1/RIP3/MLKL activation (60). Thus, EVs may regulate necroptosis through molecules like miR-412-3p targeting TEAD1, highlighting a dual role in tumor progression and organ injury.

## 3 Application of MSC-EVs in kidney diseases

Currently, preclinical studies have extensively confirmed that MSC-EVs demonstrate significant therapeutic efficacy in various kidney diseases. The underlying mechanisms primarily include delivering functional miRNAs to inhibit apoptosis, necroptosis, and inflammatory responses; Enhancing autophagy, promoting cellular repair and survival; Modulating metabolic pathways, improving energy homeostasis and oxidative stress; Regulating immune responses (e.g., increasing Tregs, inhibiting Th17/STAT3 pathway), mitigating immune-mediated renal injury (**Table 1**).

**Table 1:** Representative MSC-EVs in renal treatment.

Model	Cell type	Cargo	Method	Outcome
IRI mouse	BMSCs, UC-MSCs	TFAM mRNA and mtDNA	6.96 × 10^10^ particles, intravenous injection, at 4 h after reperfusion	Restored TFAM to attenuate mitochondrial damage and inflammation
IRI mouse	UC-MSCs	miR-125b-5p	100 ug,intravenous injection,at day 0 and 1 after reperfusion	Promoted tubular repair by targeting cell cycle arrest and apoptosis through miR-125b-5p/p53 pathway
5/6 SNx rats	BMSCs		150 μg/week, intravenous injection, for 16 weeks after SNx	downregulated Smurf2 and upregulated Smad7 expression Reduced RF
RVD pigs	AMSCs		100 ug,intrarenal arterial infusion	Preserved renal hemodynamics and function, attenuated renal injury
UUO mouse	PSC-MSCs		1×10^11^particles, intravenous injection	Upregulated SIRT6 and downregulated β-catenin and its downstream targets (PAI-1, Fsp1, Axin2) to prevent injury
Renal anemia mouse	Kidney MSCs		2 × 10^7^ particles, intravenous injection	Transfered human EPO mRNA into kidney tissue to prevent fibrotic and anti-inflammatory actions in the kidney
DKD mouse	UC-MSCs	miR-23a-3p	100 ug, intravenous injection	Alleviated RF and inflammation by targeting KLF3/STAT3
CLP mouse	BMSCs	miR-223-3p	100 ug, intravenous injection	Reduced inflammation, pyroptosis, kidney injury by silencing of SNRK
Ccisplatin-induced AKI mouse	BMSCs	let-7b-5p	100 ug, intravenous injection, 24 hours after cisplatin injection	Decreased apoptosis via inhibition of p53 pathway activation
IRI mouse	UC-MSCs	miR-181b-5p, miR-199b-5p, miR-126-3p, miR-378-5p, etc	1×10^10^ particles, intravenous injection, at 0, 24, and 48 hours after reperfusion	Improved renal function, prevented kidney injury
DKD mouse	UC-MSCs	CK1δ and β-TRCP	10 mg/kg, intravenous injection, once every 3 days	Attenuates RF by inhibition of TGF-b1/Smad2/3/YAP signaling
UUO mouse	BMSCs	miR-181d	20 μg/mL, intravenous injection	Attenuated RF by targets KLF6 and NF-κB
MRL/lpr mouse	UC-MSCs		100 ug, intravenous injection	Inhibited IL-6/STAT3/IL-17A pathway to reduce glomerulosclerosis, interstitial infiltration, and perivascular inflammation

systemic lupus erythematosus (SLE), ischemia-reperfusion injury (IRI), unilateral ureteral obstruction (UUO), diabetic kidney disease (DKD), cecal ligation and puncture (CLP), bone marrow-derived mesenchymal stem cells (BMSCs), umbilical cord-derived mesenchymal stem cells (UC-MSCs), adipose-derived mesenchymal stem cells (AMSCs), mitochondrial transcription factor A (TFAM), renovascular disease (RVD), pluripotent stem cell-derived MSCs (PSC-MSCs).

### 3.1 AKI

AKI is characterized clinically mainly by elevated serum creatinine levels and reduced urine output, and it is a condition closely associated with high morbidity and mortality (1). Its pathogenesis involves direct toxic effects of various triggers on renal TECs, which subsequently lead to intrarenal hemodynamic disturbances and the deposition of metabolic products in the kidneys. Moreover, these factors can activate immune cell-mediated inflammatory responses and exacerbate oxidative stress, potentially progressing to CKD (61). Current treatments for AKI primarily include hemodialysis and kidney transplantation; however, these approaches often fail to achieve fundamental recovery of renal function. In recent years, MSC-EVs have showed great potential in treating AKI due to their ability in anti-inflammatory and tissue repair processes.

#### 3.1.1 IRI

IRI is a common pathological process in clinical practice, characterized by aggravated cellular damage following the restoration of blood flow after a period of ischemia. IRI is recognized as one of the key factors leading to AKI, and its pathological mechanisms are complex, involving multiple processes such as oxidative stress, inflammatory cell infiltration and cytokine release, TECs apoptosis, as well as podocyte injury (62). Based on these mechanisms, recent studies have explored the therapeutic value of MSC-EVs in IRI.

MSC-EVs significantly improve renal function in mice with IRI, alleviating renal tissue necrosis, apoptosis, inflammation, and oxidative stress, with a protective effect comparable to that of the parent MSCs. Further mechanistic investigation revealed that the renoprotective effect of EVs may be associated with activation of the ERK1/2 signaling pathway (63). Using hypoxia preconditioning combined with 3D culture of UC-MSCs spheroids, it was found that these optimized conditions not only significantly increase EVs yield but also induced high expression of miR-210. These miR-210-enriched EVs effectively delivere the miRNA to renal cells, enhancing cell survival and migration under hypoxia (64). Study showed that hypoxic preconditioned MSC-EVs enhance renal recovery in IRI by restoring mitochondrial fatty acid oxidation (FAO) via upregulating CPT1A, improving mitochondrial quality and ATP production, while reducing oxidative stress and RF (65). MSC-EVs restore renal function in UUO induced AKI by lowering creatinine and normalizing white blood cells, demonstrating their therapeutic potential. Metabolomic analysis further identify six key biomarkers (including carnitine and melibiose) associated with a return to a healthy state, pointing to novel treatment targets (66).

#### 3.1.2 Nephrotoxic AKI

Nephrotoxic AKI manifests as a rapid deterioration of renal function, which is directly or indirectly caused by various nephrotoxic substances. Common pathogenic factors include medications (such as chemotherapeutic agents, nonsteroidal anti-inflammatory drugs, and contrast media), environmental toxins, or endogenous toxins (for example, myoglobin released in rhabdomyolysis). The mechanisms of nephrotoxic AKI primarily involve direct damage to TECs, induction of intrarenal vasoconstriction, or causation of tubular obstruction. Among the agents capable of inducing AKI models, cisplatin and glycerol are widely utilized (67).

The therapeutic potential of MSC-EVs in AKI is further demonstrated through their delivery of specific miRNAs that precisely regulate distinct cell death pathways and inflammatory responses. For example, miR-1184 is downregulated in AKI, and its delivery via MSC-EVs effectively alleviate cisplatin-induced TECs injury. Mechanistically, miR-1184 directly targets and inhibits the transcription factor FOXO4, thereby modulating downstream signaling pathways, which ultimately suppresses apoptosis and the inflammatory response (IL-1β/TNF-α) and induces G1 phase cell cycle arrest (68). Another study found that aged mice are more vulnerable to cisplatin-induced AKI, with increased DNA damage, p53 signaling, and apoptosis in kidneys. Mechanistically, MSC-EVs deliver miRNAs, notably let-7b-5p, which is downregulated in aged kidneys but restored by MSC treatment. Let-7b-5p alleviate DNA damage and apoptosis in TECs by inhibiting p53 (69). It was also discovered that UC-MSC-EVs alleviate kidney injury by delivering miR-874-3p, which inhibits RIPK1 and downstream PGAM5 to suppress necroptosis, while human UC-MSC-Exos promote Drp1 dephosphorylation at Ser637 to restrain mitochondrial fission and maintain homeostasis. These mechanisms collectively prevent AKI progression to CKD (70). Furthermore, platelet-rich plasma (PRP)-stimulated MSCs (PRP-MSCs) enhance EVs release via AKT/Rab27 pathway activation, inhibiting renal tubular apoptosis and promoting tissue repair through paracrine mechanisms. This highlights PRP preconditioning as a strategy to optimize MSC-based therapies by boosting paracrine function (71).

### 3.2 CKD

#### 3.2.1 Renal fibrosis

Renal fibrosis (RF) is the common pathological outcome of various kidney diseases, characterized by excessive accumulation of ECM in the glomerular and interstitial areas. The main pathological features of RF include inflammatory cell infiltration, activation and proliferation of fibroblasts, ECM deposition, renal tubular injury, and microvascular rarefaction. The primary focus of CKD treatment is the management of prevalent risk factors like hypertension, diabetes, relying on pharmacological agents (72). Although these treatments slow down disease progression, they are associated with certain side effects. In recent years, MSC-EVs have demonstrated potential in treating RF.

MSC-EVs combat RF through interventions in fundamental pathological mechanisms. It was found that BMSC-EVs alleviate RF and improve function in a 5/6 nephrectomy (SNx) rat model by modulating the Smurf2/Smad7 axis (73). Further research demonstrate that, In a rat menopausal model with ovariectomy-induced CKD, renal impairment, oxidative stress, inflammation, and fibrosis are observed. Both BMSCs and their EVs reverse these changes and restore renal structure and function. EVs are more effective than BMSCs in reducing inflammation, highlighting the potential of cell-free therapy for CKD (74). BMSC-EVs exert anti-fibrotic effects in RF via their carried miR-181d. Mechanistic studies revealed that miR-181d directly targets and downregulates KLF6, thereby inhibiting NF-κB signaling pathway. MiR-181d-enriched BMSC-EVs effectively reduce fibrosis markers, confirming their role in alleviating RF progression through this molecular mechanism. MSC-EVs ameliorate UUO-induced RF by suppressing aerobic glycolysis in TECs. Mechanistically, MSC-EVs derived miR-21a-5p directly targets and inhibits PFKM, a key glycolysis rate-limiting enzyme, thereby attenuating glycolytic flux. This highlights the miR-21a-5p/PFKM axis as a key mechanism in MSC-EVs–mediated metabolic suppression (75). These findings collectively demonstrate the multi-faceted efficacy of MSC-EVs against RF.

#### 3.2.2 Diabetic kidney disease

DKD is a common chronic microvascular complication of diabetes and one of the major causes of CKD (76). Due to its complex metabolic disturbances, DKD often becomes more challenging to treat than other kidney diseases once it progresses to ESRD (77). Therefore, timely prevention and management are of great significance in delaying the progression of DKD.

MSC-EVs have been shown to reduce proteinuria and glomerulosclerosis by modulating glucose metabolism and suppressing inflammatory responses. MSC-EVs alleviate DKD by delivering miR-125b, which targets TRAF6 to inhibit apoptosis via Akt signaling activation and enhance autophagy (78). Another study showed that BMSC-EVs alleviate DKD manifestations, including hyperglycemia, renal dysfunction, dyslipidemia, and pathological renal damage, potentially by inhibiting JAK2/STAT3 overactivation (79). Moreover, EVs not only significantly improve renal function and attenuate renal pathological injury in SNx CKD rats, but their protective mechanism is also associated with upregulating promoter activity and protein expression of the klotho gene in the kidney (80). Meanwhile, human placental MSCs-EVs alleviate RF and improve renal function in diabetic models via the miR-99b-5p/mTOR/autophagy axis, which offers a cell-free therapy for DKD (81).

Also, UC-MSC-EVs reduce blood glucose, improve lipid metabolism and liver function, and alleviate tissue damage in mice. *In vitro*, they enhance glucose uptake in insulin-resistant cells and protecte β-cells from oxidative damage by reducing ROS and inhibiting apoptosis. Mechanistically, EV-enriched miR-191-5p targets DAPK1, activating the PI3K/AKT pathway and upregulating Nrf2 and SOD1 (82). In addition, another study found that MSC-EVs intervention in DKD mice markedly suppress the NOD2 pathway and its downstream protein expression, leading to decreased levels of inflammatory cytokines, thereby mitigating podocyte dysfunction (83). Thus, MSC-EVs ameliorate diabetic conditions via direct metabolic improvement, anti-inflammatory actions, and cellular protection.

### 3.3 Other kidney diseases

Lupus nephritis (LN) is one of the most common and severe complications of systemic lupus erythematosus (SLE). It is characterized by inflammation and damage resulting from abnormal autoimmune attacks on the kidneys. Its primary clinical manifestations include proteinuria, hematuria, and renal dysfunction, which are critical factors influencing the prognosis of SLE patients (84). The treatment aims to control renal inflammation, delay disease progression, and minimize drug-related side effects (85). In LN, MSC-EVs can modulate autoimmune responses and reduce immune complex deposition. Specifically, human UC-MSC-EVs ameliorate SLE in MRL/lpr mice by reducing proteinuria, renal damage, splenomegaly, anti-dsDNA IgG, and mortality. Mechanistically, they modulate T cell responses: decreasing Th1, DNT, and renal Th17 infiltration while boosting Tregs, thereby lowering IFN-γand IL-6. This is mediated via suppressed STAT3 activation and downregulated renal IL-17A (86). These findings demonstrate that MSC-EVs can effectively alleviate LN progression by restoring immune balance and mitigating renal injury.

Renal anemia represents a major complication of CKD, notable for its high prevalence, primarily induced by insufficient erythropoietin (EPO) following kidney damage, which leads to reduced erythropoiesis and decreased hemoglobin levels. A work found that engineered EPO(+)-MSCs derived from kidney can efficiently secrete EPO and yield EVs carrying EPO mRNA, which are capable of delivering functional EPO mRNA to target cells. Furthermore, *in vivo* experiments demonstrated that intraperitoneal injection of either EPO(+)-MSCs or their derived EPO(+)-EVs into CKD mice for two weeks significantly increase hemoglobin levels and improve renal function. This research confirms that genetically engineered EPO(+)-EVs can effectively alleviate renal anemia, highlighting their considerable potential as a novel cell-free therapeutic strategy (87).

## 4 Engineering strategies for MSC-EVs

Building on the promising therapeutic outcomes of MSC-EVs in diseases, these vesicles have garnered increasing attention as versatile platforms for drug delivery in biomedicine (88). However, native MSC-EVs still face significant limitations in practical applications. Firstly, insufficient targeting specificity and lack of active recognition capability for specific diseased cells or tissues, leading to nonspecific distribution *in vivo*, which reduces enrichment efficiency at lesion sites and may increase off-target risks. Secondly, difficulty in precise regulation of therapeutic cargo, as the types and abundance of active components in naturally secreted EVs exhibit heterogeneity, affecting the controllability and consistency of therapeutic efficacy. Consequently, research efforts are increasingly directed toward addressing their inherent limitations through engineered modifications, with the goal of developing more refined treatment modalities (89). Current engineering strategies primarily involve the deliberate manipulation of EVs cargos or surface characteristics using physical, chemical, or genetic techniques.

**Figure 2:**
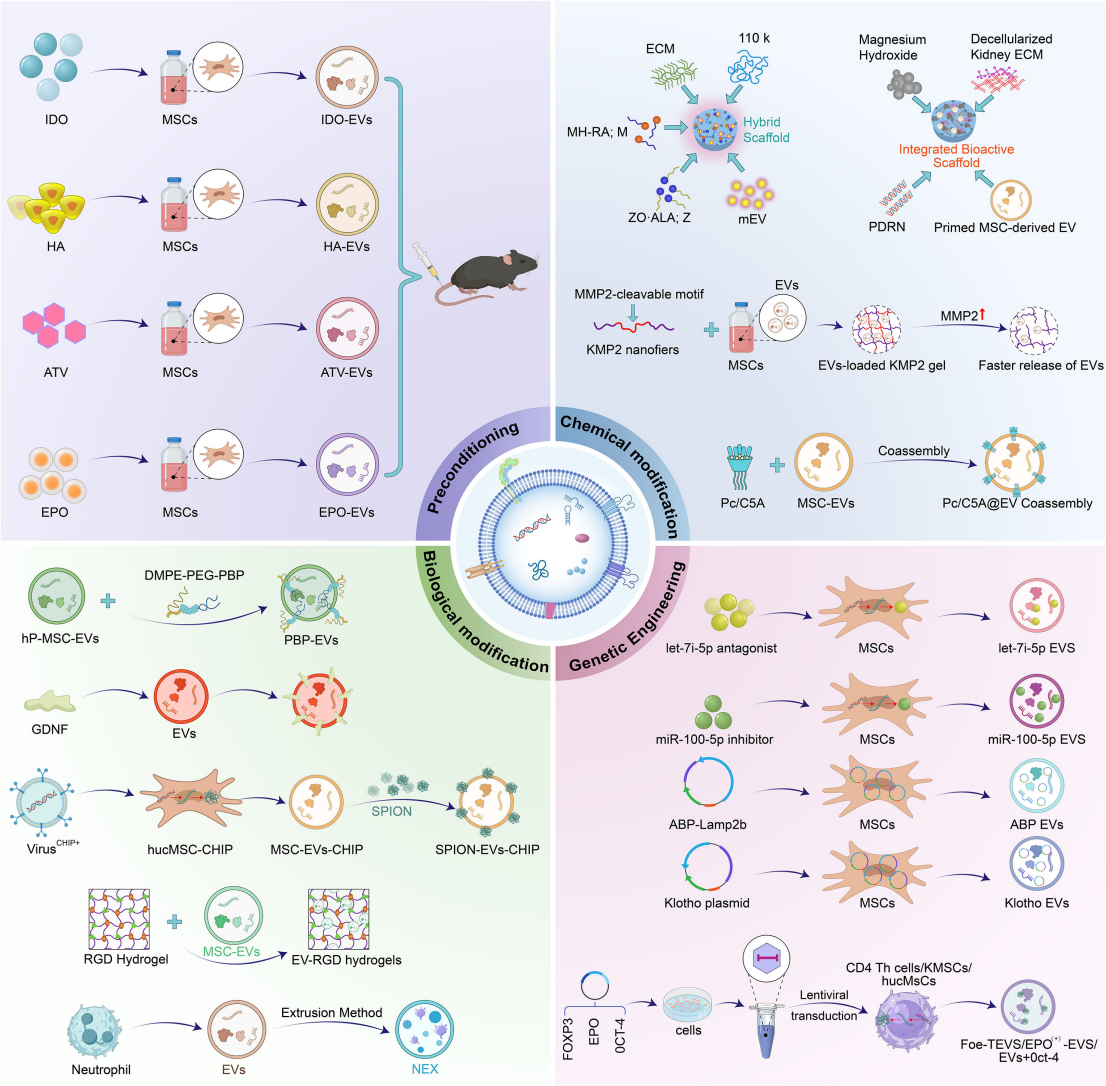
Engineering strategies for MSC-EVs. Hyaluronic acid (HA), Erythropoietin (EPO), albumin-binding peptides (ABP), indoleamine 2,3-dioxygenase (IDO), Atorvastatin (ATV), P-selectin-binding peptide (PBP), carboxyl terminus of Hsc70-interacting protein (CHIP), glial cell line-derived neurotrophic factor (GDNF), polydeoxyribonucleotide (PDRN).

To address the issue of insufficient targeting specificity, the primary strategies include: Modifying the surface of EVs with specific targeting molecules (such as antibodies, peptides, or aptamers) to enhance their binding affinity to receptors at pathological sites; Achieving externally controlled targeted enrichment through physical methods like magnetic field response or ultrasound guidance; Utilizing engineered parent cells to incorporate targeting modules during the biogenesis phase of EVs. For therapeutic cargo regulation, customized loading of therapeutic components is achieved through genetic editing of parent cells or by loading drugs/nucleic acids into isolated EVs. Such modifications enable enhanced targeting precision to specific cells or tissues, a crucial feature for spatially controlled therapies. Beyond improving targeting accuracy, engineering approaches significantly broaden the functional scope of MSC-EVs and introduce complementary therapeutic enhancements. The capacity to achieve site-specific delivery while reducing non-specific effects represents a particular advantage of these optimized systems. These developments hold considerable clinical relevance, paving the way for the use of engineered MSC-EVs as next-generation therapeutics capable of providing customized treatment with superior efficacy and minimized risks (90) (**Figure 2**).

A range of technologies are now being utilized for the engineering of EVs, with the goal of improving their stability, targeting accuracy, and delivery efficiency to pathological sites (91). To improve therapeutic outcomes, researchers have developed multiple engineered MSC-EVs strategies, which can be categorized into two approaches: engineering the parent cells and directly modifying the MSC-EVs (92). The former involves genetically or chemically modifying MSCs during the biogenesis phase of MSC-EVs, enabling the cells to produce vesicles carrying target molecules (such as proteins or nucleic acids) or possessing specific surface properties. In contrast, the latter approach involves directly manipulating MSC-EVs after they have been secreted by the cells. Although this method avoids complex genetic manipulation of cells, it may affect the integrity and bioactivity of the MSC-EVs (**Table 2**).

**Table 2:** Representative MSC-EVs-mediated drug delivery for kidney diseases.

Model	Cell type	Cargo	Loading method	Surface modification	Therapeutic method	Outcome
Cisplatin-induced AKI mouse	AMSCs			P-HA	Intravenous injection on Day 2 and 3 post-AKI induction	Reduced TECs apoptosis and decreased M1 macrophages
UUO mouse	BMSCs	let-7i-5p antagomir	Generation of let-7i-5p antagomir induced MSCs		50 ug, intravenous injection, twice a week post UUO for 4 weeks	Reduced RF via activating TSC1/mTOR pathway
UUO mouse	AMSCs	miR-126–5p		DSPE-PEG-RGD	200 ul, intravenous injection	Upregulated SIRT1 via m6A methylation mediated by miR-126–5p/ALKBH5 axis to reduce renal injury
UUO mouse	AMSCs	GDNF	Generation of GDNF transfected MSCs		150 μl, intravenous injection	Improved peritubular capillary density and reduced RF by activated PI3K/Akt/eNOS
IRI mouse	BMSCS	indoleamine 2,3-dioxygenase (IDO)	Generation of -overexpressed MSCs		100 ug single intravenous injection,6 hours after IRI	Accelerated recovery of renal function and enhanced M2 macrophage polarization
IRI mouse	UC-MSCs	miR-100-5p	Generation of miR-100-5p mimic or inhibitor transfected MSCs		5 × 10^10^ particles	Reduced tubular damage by targeting FKBP5 and cleaved caspase-3 expression via AKT
Cisplatin-induced AKI mouse	UC-MSCs	miR-125-5p, miR-146a-5p etc	Generation of neutrophil membrane-engineered MSCs		100 ug, intravenous injection, administered for 2 consecutive days starting 72 hours after cisplatin injection	Reduced apoptosis by increasing PCNA to reduce pro-inflammatory cytokines
UUO mouse	MSCs	CHIP (Carboxyl terminus of Hsc70-interacting protein)	Generation of CHIP-overexpressed MSCs		100 ug, intravenous injection	Promoted Smad2/3 ubiquitination and degradation to inhibit RF
Rhabdomyolysis-induced AKI mouse	Pluripotent stem cell-derived MSCs (PSC-MSCs	Klotho	Generation of Klotho-overexpressed MSCs		200 ul intravenous injection	Inhibited renal injury by restoration of endogenous Klotho to activate mTOR and MEK1/2
DKD rats	BMSCs	miR-221-3p	Generation of Atorvastatin (ATV) preconditioned MSCs		50 ug/ml, multiple subcutaneous injections around the wound (six points)	Accelerated wound closure, increased re-epithelialization, and enhanced collagen deposition by AKT/eNOS
5/6 (SNx) rats	UC-MSCs		Polyethylenimine (PEI) coated on PMEZ scaffolds to enhance EV immobilization		1×10^8^/ml	Regenerated kidney tissue and restorated renal functional

### 4.1 Engineering donor cells

#### 4.1.1 Genetic editing

Genetic sequences encoding target proteins—such as targeting peptides, cytokines, or signaling proteins or small RNAs like miRNAs and shRNAs are introduced into donor cells. This process leverages innate synthetic machinery to preload MSC-EVs with therapeutic cargo or surface modifications prior to their release. For example, Yang et al. developed multifunctional FOXP3-engineered EVs, which through limited exogenous gene transduction. Their surface expression include adhesion molecules and co-inhibitory receptors (notably CTLA-4 and PD-1), accompanied by the production of regulatory cytokines. These EVs effectively adhere to injured areas of renal tubules, endothelium, and glomeruli in kidney allografts, thereby mitigating local cell death and chronic fibrotic transition. Notably, transcriptome engineering restores phenotype and increases the stagnation coefficient by 2.16-fold. In mouse allograft models, EVs restore renal function, improve ultrastructural organization and glomerular filtration rate, and extend recipient survival (93).

The renoprotective effects of EVs are being amplified by targeting specific molecular pathways involved in cell survival and fibrosis. A study engineer EVs with albumin-binding peptides (ABP) to leverage endogenous albumin for enhanced drug delivery to injured kidneys, where they improve retention via macroglobulin mediated endocytosis and demonstrat superior anti-apoptotic and anti-inflammatory efficacy (94). Researchers deliver a let-7i-5p inhibitor via EVs into cellular and mouse models. Results indicate that inhibiting let-7i-5p upregulate its target gene TSC1, activating the TSC1/mTOR signaling pathway, which ultimately attenuate EMT and ECM deposition, thereby significantly alleviating RF (95). Also, enrichment of miR-100-5p in UC-MSC-EVs targets FKBP5, increases AKT phosphorylation at Ser473 (p-AKT Ser473), and inhibites apoptosis in HK-2 cells. These effects were markedly reversed when EVs were extracted from MSCs transfected with a miR-100-5p inhibitor (96). Klotho-overexpressing MSCs produces Klotho-EVs, which accumulate in injured kidneys and accelerate recovery more effectively than standard EVs. They improve renal function and structure, promote cell proliferation, and reduce injury and inflammation. Klotho-EVs uniquely activate mTOR signaling and carry a distinct cargo of protective proteins and miRNAs (80). Collectively, these studies highlight the precision and enhanced power of engineered or specifically sourced EVs in treating kidney injury.

#### 4.1.2 Culture condition engineering (preconditioning)

Genetically engineered MSC-Exos, particularly IDO-overexpressing ones (MSCs-Exo-IDO), represent a superior strategy for complete kidney repair after IRI. They are taken up by renal cells and promote anti-inflammatory M2 macrophage polarization, thereby improving the local microenvironment, accelerating functional recovery, and inhibiting apoptosis and fibrosis more effectively than conventional Exos. Engineered small EVs (pSEVs), derived from polyethylene glycolylated HA-modified MSCs, target injured kidneys via HA-CD44/TLR4 interactions. They demonstrate renoprotective and anti-inflammatory effects by inhibiting TECs apoptosis and pro-inflammatory macrophage polarization, thereby ameliorating AKI symptoms and functional markers (97). Atorvastatin (ATV) preconditioned BMSC-exos (ATV-Exos), although similar in morphology and concentration to conventional Exos, significantly enhance wound healing and angiogenesis in diabetic rats by promoting human umbilical vein endothelial cells (HUVECs) proliferation, migration, and tube formation via the miR-221-3p/AKT/eNOS pathway, without inducing toxicity (98). In a surgically constructed CKD mouse model with renal anemia, EPO(+)-EVs isolated from engineered cells effectively deliver EPO mRNA to target cells. After two weeks of intraperitoneal injection, EPO(+)-EVs significantly increas hemoglobin levels and improve renal function (serum creatinine), demonstrating potential as a novel treatment strategy for renal anemia in CKD. These advanced EV-based platforms demonstrate superior targeting and regenerative capabilities over their native counterparts (99).

### 4.2 Direct modification of MSC-EVs

#### 4.2.1 Biological modification

The surface modification of EVs with targeting peptides, such as RGD and LyP-1, via chemical methods can enhance their accumulation in the kidneys. Researchers have developed P-selectin-binding peptide (PBP)-engineered EVs (PBP-EVs) in AKI. PBP-EVs exhibit selective targeting toward injured kidneys, while also enabling molecular imaging to quantify P-selectin expression in renal tissue, thereby providing spatiotemporal information for early AKI diagnosis. Moreover, PBP-EVs demonstrate remarkable renal protective effects by promoting kidney repair and suppressing RF, which include reduction of inflammatory infiltration, an enhancement of reparative angiogenesis, and suppression of maladaptive renal parenchymal repair (100). Similarly, by modifying EVs with RGD peptides, their targeting ability to hypoxic renal tissue is significantly improved. These modified EVs deliver miR-126-5p, which stabilizes SIRT1 expression through m6A RNA modification, thereby restoring key molecular functions. This approach further promotes vascular health and delays RF (101).

A study developed a CKD treatment platform using SPION-decorated MSC-EVs overexpressing carboxyl terminus of Hsc70-interacting protein (CHIP) (SPION-EVs). This system enables magnetic field-directed accumulation in injured kidneys, enhancing renal targeting. The delivered CHIP induces degradation of Smad2/3 in TECs, thereby attenuating Smad2/3-mediated fibrosis and collagen deposition, offering a promising nanoplatform for RF (102). Wu et al. constructed engineered hybrid vesicles (NEX) by fusing human UC-MSC-EVs with human neutrophil membrane vesicles. Their results demonstrated that NEX significantly enhances targeting ability to injured renal tissue and effectively improves renal function by reducing pro-inflammatory factor expression, lowering oxidative stress, inhibiting renal cell apoptosis, and promoting cell proliferation. Additionally, NEX was shown to regulate the uptake of EVs by different cell types (103). Moreover, glial cell line-derived neurotrophic factor (GDNF)-modified AMSCs enhance growth factor secretion, boosting angiogenesis and endothelial repair. In UUO models, they exhibit superior homing, alleviating microvascular rarefaction, fibrosis, hypoxia, and oxidative stress by activating the PI3K/Akt/eNOS pathway and suppressing EMT.

#### 4.2.2 Chemical modification

By co-assembling a hypoxia-sensitive macrocycle (C5A), a dye (Pc), and MSC-EVs, Cheng et al. constructed Pc/C5A-EVs. This nano-platform leverages MSC-EVs targeting to achieve precise near-infrared imaging of hypoxic kidneys. It further treats renal injury by inhibiting the HIF-1α/NF-κB pathway, reducing tubular cell apoptosis, and promoting macrophage polarization, thus presenting an innovative theranostic strategy with broad clinical potential (104). To prevent the rapid clearance of MSC-EVs, an MMP2-sensitive hydrogel (KMP2) is developed for encapsulation and sustained release. In IRI, KMP2-EVs surpass free EVs in improving kidney function by inhibiting apoptosis and inflammation, promoting angiogenesis, and mitigating chronic RF (105). Also, a porous PLGA/Mg(OH)_2_/kidney ECM scaffold, functionalized with polydeoxyribonucleotide (PDRN) and TNF-α/IFN-γ-primed MSC-EVs (TI-EVs), synergistically enhance proliferation and angiogenesis while reducing fibrosis and inflammation, promoting glomerular regeneration in a partial nephrectomy model (106). Additionally, Rhim et al. develope a biodegradable porous PLGA scaffold (PMEZ/mEV) integrated with Mg(OH)_2_, ECM, ZnO, and MSC-EVs. This scaffold synergistically promotekidney repair in SNx models by sustaining nitric oxide release and enhancing regenerative functions (107). These engineered systems demonstrate that enhancing EV delivery and retention can synergistically amplify their inherent regenerative capabilities.

The complexity of kidney diseases, driven by multiple cell types, presents a challenge for EVs-based therapies that require precise cellular targeting and pathway modulation. To improve efficacy, MSC-EVs can be engineered to target multiple pathological factors or used in combination therapies, potentially yielding superior results over single treatments (108). However, key challenges remain, including standardizing isolation and characterization methods, ensuring quality in large-scale production, determining optimal doses, and evaluating long-term safety. Future research should focus on establishing GMP production standards, optimizing engineering strategies, exploring combination therapies, and advancing clinical translation (109).

The key point of this review lies in achieving a paradigm shift in research perspective: systematically defining MSC-EVs as “engineerable multifunctional therapeutic platforms” rather than merely biological effect carriers. By systematically constructing this new paradigm, we have not only expanded the understanding of cutting-edge mechanisms but also comprehensively delineated the full spectrum of engineering strategies from donor cell modification to direct vesicle manipulation. Building upon this framework, we explicitly propose that the future trajectory of the field lies in achieving precise targeting through rational design and constructing combination therapies via synergistic integration, thereby providing a clear research and development roadmap for advancing MSC-EVs from fundamental studies to next-generation intelligent therapies for kidney diseases (110, 111).

## 5 Conclusion

In summary, the increasing incidence of kidney diseases represents a critical and unmet medical need, underscoring the imperative for novel therapeutic development. MSC-EVs have demonstrated considerable potential for treating kidney diseases through xeno-free, multi-target and multi-pathway characteristics, making them a research focus in regenerative medicine. With advances in in-depth research into the therapeutic mechanisms, engineering modification technologies and progress in clinical translation, MSC-EVs are expected to emerge as a novel therapeutic option for kidney diseases. Moving forward, more high-quality clinical studies are needed to verify their long-term safety and efficacy, thereby facilitating the translation of this innovative therapy into patient benefit.
